# Measurements of sky brightness at Bosscha Observatory, Indonesia

**DOI:** 10.1016/j.heliyon.2020.e04635

**Published:** 2020-08-19

**Authors:** D. Herdiwijaya, R. Satyaningsih, Luthfiandari, H.A. Prastyo, E.P. Arumaningtyas, M. Sulaeman, A. Setiawan, Y. Yulianti

**Affiliations:** a Astronomy Department, Bandung Institute of Technology, Ganesha 10 Bandung 40132, Indonesia; b Bosscha Observatory, Bandung Institute of Technology, Peneropongan Bintang, Lembang 40391, Indonesia; c Center for Research and Development, Indonesian Agency for Meteorology, Climatology, and Geophysics, Angkasa I-2, Kemayoran, Jakarta 10720, Indonesia; d Bandung Geophysics Station, Indonesian Agency for Meteorology, Climatology, and Geophysics, Cemara 66 Bandung 40161, Indonesia

**Keywords:** Astronomy, Atmospheric science, Light pollution, Night sky brightness, Meteorological conditions, Astronomical observation

## Abstract

To determine the level of light pollution due to human activities, we performed sky-brightness measurements at Bosscha Observatory, Indonesia (107°36′E; 6°49′S, 1300 m above sea level) for seven years from 2011 to 2018, using a portable photometer pointed at the zenith. From 1692 nightly records, we found that the average brightness on moonless nights reached the 19.70 ± 0.84 and 19.01 ± 0.88 astronomical magnitudes per square arcsecond (mpass), with median values of 19.73 mpass and 19.03 mpass for the AM and PM periods, respectively. The darkest skies occurred in the peak of the summer season during the month of July, which corresponds to the lowest annual temperature, precipitation, and relative humidity. The internal temperature of our Sky Quality Meter is adequately stable, and our results correlate well with other measurements. The sky brightness depends on the age of the Moon (days past new Moon) and on seasonal monthly variations, but it is not related to the lunar distance. The night-SB quality can be modified by the coupled climate system as a diurnal cycle to an 11-year solar cycle. The cities around the Observatory, Bandung and Lembang, clearly make strong contributions to light pollution in the area due to unshielded light sources.

## 1 Introduction

Bosscha Observatory (107°36′E; 6°49′S, 1300 m above sea level, IAU observatory code 299), which has the first modern telescopes in Indonesia, is located in the city of Lembang, West Java. It is on the north side of the ancient plateau of Bandung Basin, a high-density populated area, and in the southern part of the Tangkuban Perahu volcano. The Observatory is also near the active 29-km-long Lembang fault, which has a slip rate of 3 mm/year (Daryono et al., 2019; Dam and Suparan, 1992). The Observatory was named after Karel Albert Rudolf Bosscha from the Netherlands, who mostly funded and built it from 1923 to 1928 (Voute, 1933). The geomorphology of the Bandung Basin plays an important role in the diurnal cycle of near-surface climate parameters (Geiger et al., 2003). The complex geomorphological processes and anthropogenic structures caused by humans in urban areas influence the formation and channeling of the local wind route circulation causing flow and pollutant dispersion (Fernando et al., 2010). At night, outdoor lighting from an unshielded light fixture causes a man-made sky glow. All these factors have become an integrated part of environmental management (Scherer et al., 1999; Suder and Szymanowski, 2014).

Moreover, economic and population growth in the nearby cities have caused sky glow to spread out over the night sky above the Observatory (Malasan et al., 2001). Light pollution is growing rapidly due to human economic activity in the main city, and the sky glows interfere significantly with astronomical research. The elusive effects of uncontrolled and unshielded outdoor lighting produce light trespass and glare, affecting human health and wildlife, increasing environmental degeneracy, and driving high-cost economic impacts (Rich and Longcore, 2004; Gallaway et al., 2010; Falchi et al., 2011; Haim and Zubidat, 2015; Cho et al., 2015; Touitou et al., 2017; Irwin, 2018).

At the Bosscha Observatory (BO), sky-brightness measurements were previously carried out using CCDs with narrow-band Johnson V filters (Bessel, 2005), yielding an average value on July 6–9, 2011 of 16.49 ± 0.92 astronomical magnitudes per square arcsecond (mpass) at zenith angles *z* between 23°–74° (Azzahidi et al., 2011). Unfortunately, there has been no continuous sky-brightness monitoring program with high-cadence data recording at the BO. Instead, we have measured the night-sky brightness systematically using Unihedron's Sky Quality Meter (SQM), a portable photometer with a broadband filter, for seven years from 2011 to 2012 and 2015 to 2018. It is also useful to examine the sky brightness before and after midnight, which is helpful for optimizing successful nighttime observations (Voute, 1933). Instead, of semi-diurnal sky-brightness variations, a climatic cycle of an 11-year solar activity has been reported from several astronomical sites, which we will also address herein.

## 2 Methods

The portable and low-cost SQM photometer is a silicon photodiode sensor combined with a linear-response light-to-frequency converter that has adequate stability and accuracy (den Outer et al., 2011; Schnitt et al., 2013; Sánchez de Miguel et al., 2017). It can be operated over the wide temperature range from −25 °C up to 70 °C, and the output frequency response is constant over the range from 15 °C to 55 °C. This is useful in the equatorial region, which has stable annual temperatures. The temperature-compensated sensor is covered with a broadband visual filter having more than 70% light transmission in the wavelength range from 350 to 580 nm, peaking at 500 nm.

An integral lens produces a narrow, cone-shaped field of view, with an angular sensitivity having a full width at half maximum of about 20° (Cinzano, 2005). The output of the SQM gives the sky brightness in mpass. We tested the angle of incidence of the SQM using a simple method with a variable-wattage dimmer lamp in order to determine the angle of acceptance and the linear response of the device. We confirm that the device captures incoming light within a 20°-cone shape. The measured lamp brightness in mpass units decreased with increasing power to the lamp, in the same way that astronomical magnitudes become smaller as sources become brighter. We changed the brightness to a linear scale in nano-Lambert units and found a good linear response, with the goodness of fit corresponding to an R-squared value of 0.97 (Pravettoni et al., 2016).

We connected the device to a laptop *via* a USB extension cable of about 5 m long. We oriented the SQM toward the zenith and set it to record continuously from sunset to sunrise at 3–5 s intervals. We used a white PVC pipe and capped it with a UV-protective glass filter. We used a silicone seal around the filter to prevent water from seeping in. We checked the device routinely and cleaned the glass cover every three months for the offset correction value owing to its clarity. We have used four SQMs for this study, and we examined the offset value every time we changed the device. For the output data, we checked within 2 min period consisting of 24–40 data, and we neglected data with highly different readings for the internal temperature (>3 °C) or brightness (>1 mpass) that might be due to lightning flashes. The duration of this abnormal brightness condition can last up to 2 min. After removing these anomalies, we were left with 1692 nights of data covering the years from 2011 to 2018. We then divided every night into PM and AM periods and determined the average sky brightness within 4 h before (PM) and after (AM) local midnight. The solar elevation angle for these periods was less than −20° (below the horizon).

## 3 Meteorological conditions

**Figure 1:**
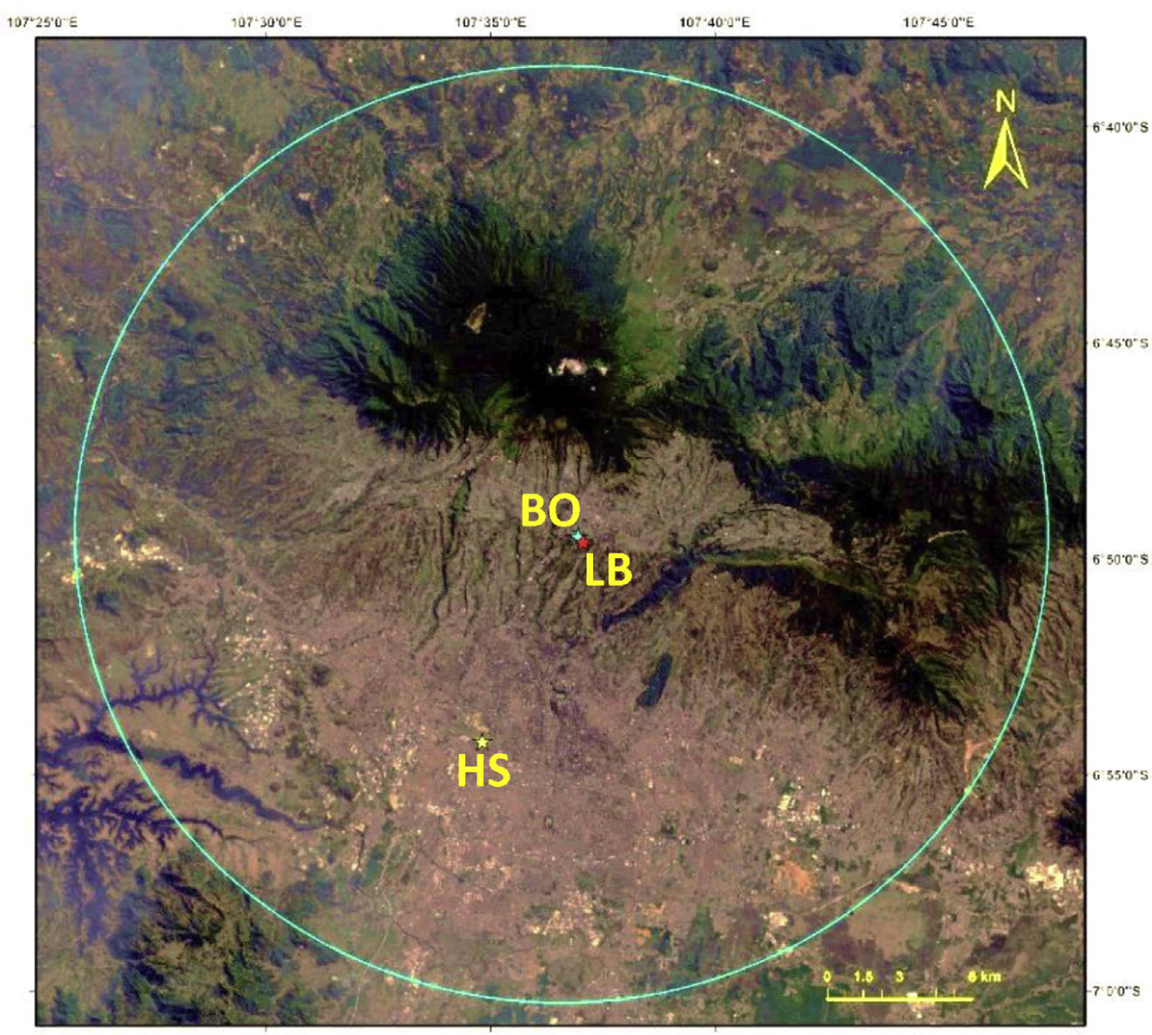
Position of the Bosscha Observatory (BO), Lembang (LB), and Hussein (HS) stations based on Landsat 8 daylight image within a 20 km radius. The northward side from the observatory is the Tangkuban Perahu volcano. The westward side is the Lembang fault. The southward side is Bandung City.

Daily meteorological data (temperature − T, precipitation, sunshine duration −SD, and relative humidity − RH) near the BO were recorded at the Lembang station (06°49′35.6″S, 107°37′03.6″E, elevation: 1241 m) of the Agency for Meteorology, Climatology, and Geophysics (BMKG) from 1980 to 2017. The temperature was recorded three times each day at 7 AM, 1 PM, and 6 PM, instead of recording the average, maximum, and minimum temperatures. The relative humidity was also recorded at these three times, together with the average humidity for the day. We also used daily-precipitation parameters from the Southeast Asian Climate Assessment and Dataset (SACA&D; http://sacad.database.bmkg.go.id) that are made available, free of charge, at the Hussein station (STAID 153, 06°52′48″S, 107°36′00″E, elevation: 791 m) from 1975 to 2012. This station is located at Bandung City, about 10 km south of the Observatory. In Figure 1, meteorological station positions overlaid on visual image obtained using Landsat-8 satellite (https://earthexplorer.usgs.gov/). Then, we compared these data with the early meteorological data at the Observatory that spanned 10 years, from 1923 to 1932, except for the precipitation data that covered 40 years (Voute, 1933).

The BMKG data for 38 years depict that all meteorological data showed relatively constant values, except for precipitation that showed a decreasing gradient of −0.22 ± 0.03 mm/year. The average, maximum, and minimum temperatures were 20.1 °C ± 0.3 °C, 25.0 °C ± 0.7 °C, and 16.3 °C ± 1.1 °C, respectively. The average relative humidity, in the morning at 7 AM and in the afternoon at 1 PM were 84.5% ± 2.2%, 88.7% ± 1.8%, and 73.1% ± 3.9%, respectively. The precipitation and the sunshine duration were 10.1 ± 3 mm and 53.4% ± 5.3%, respectively.

**Figure 2:**
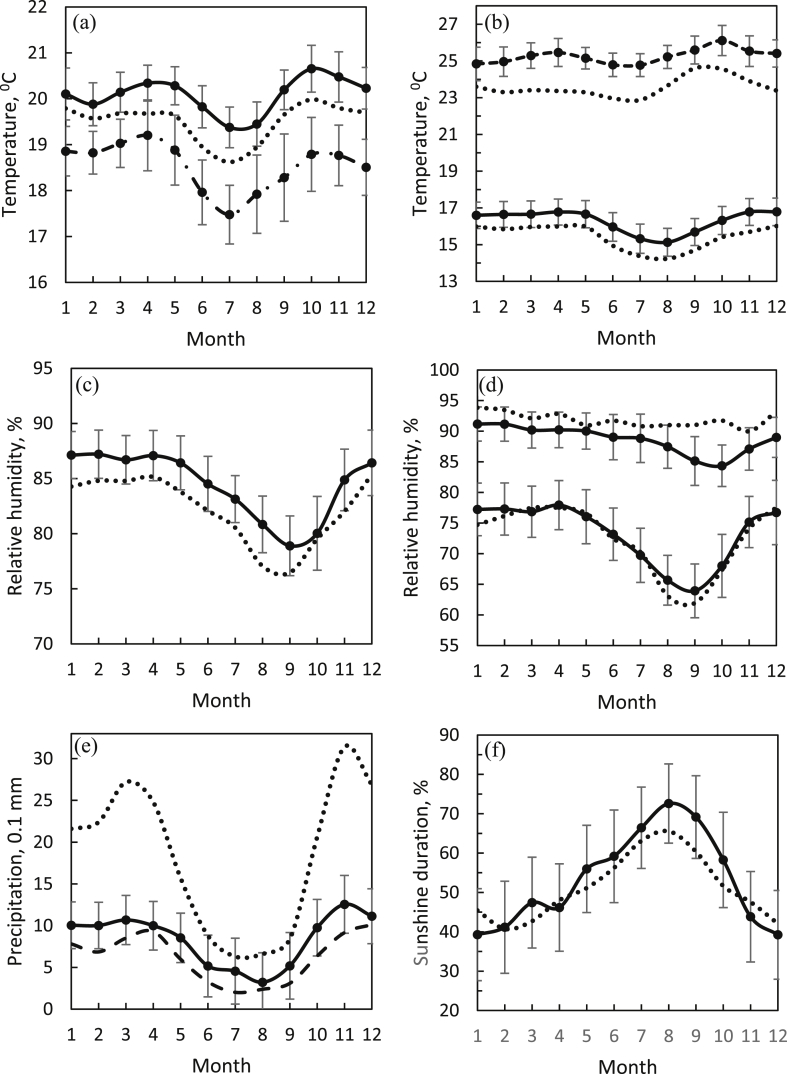
(a) Monthly temperatures: Average (solid line) from BMKG for 1980–2017, from Voute for 1923–1932 (dotted line), and from the SQM (dashed dot line). (b) Maximum and minimum temperatures from BMKG and Voute. (c) Relative humidity from BMKG and Voute. (d) Relative humidity at 7 AM (upper lines) and 1 PM (lower lines) from BMKG and Voute. (e) Precipitation intensity from BMKG, SACA&D for 1975–2012 (dashed line), and Voute. (f) Sunshine duration from BMKG and Voute.

The monthly temperature fluctuations are shown in Figure 2a and b. The peak of the dry season occurs in August, when the average temperature, precipitation, and sunshine duration are 19.4 °C ± 1.0 °C, 32 ± 17 mm, and 72.6% ± 10.1%, respectively. The average temperature remained stable from 19 °C to 21 °C. However, the maximum temperature increased to its highest point in October, about one month later than in the years 1923–1932. Moreover, the maximum temperature (September–October) in recent periods has increased by about 1.7 °C, or about 6.3% (corresponding to an overall temperature increase of about 7.2%), compared with 1923–1932. Although the minimum temperature (July–August) had the same pattern, the recent period was hotter by 0.9 °C, or about 6.4% (within two periods, annually increase of about 5.5%). The seasonal average temperature from the SQM for the seven years of this study showed the same patterns but was slightly cooler than the BMKG data (less than 1.5 °C). We, thus, confirm that the SQM temperature sensor is sufficiently reliable for long-term monitoring work.

The lowest average relative humidity occurred in September, at a value of 78.9% ± 4.2%. From January to April, the relative humidity was constant. In the past, the relative humidity in the morning was higher and more stable, at 90%–95%, than in the recent period, which showed the lowest relative humidity in September–October. The relative humidity at noon has been the same for 95 years, but recently, mornings have been about 3.5% less humid for the whole year. See Figure 2c and d.

The precipitation intensity at Bandung City from 1975 to 2012 was the same as at the Observatory from 1980 to 2017. Comparing the precipitation from the early times at the Observatory (Voute, 1933), it followed the same annual seasonal pattern with summer from June to September and the rainy season from November to March. However, at present, it is significantly dryer at approximately 100.9% annually, as shown in Figure 2e. The minimum and maximum precipitation still occur in August and November.

The average sunshine durations showed similar patterns at one sigma over the last 95 years. A noticeable increase was observed in the sunlight percentages in May, June, July, and August with a maximum at 72.6% ± 10.1%. The pattern returned to normal at approximately 40% the following month. The average annual number of daily Sun hours remained at approximately 6.4 h per day. The air temperature trend did not give evidence of a statistical significance with the temporal variation of the sunshine duration. The negative correlation expected between the sunshine duration and the relative humidity is often not clearly evident in seasonal records. Therefore, their anomaly must depend on other factors, such as changes in the total cloud cover and aerosol optical thickness. Further analysis should start by a setup of long-term data sets of the cloudiness factors. Our results showed that no local variation existed in the sunshine duration when comparing data from 1923 to 1932 and 1980 to 2017. The sunshine duration can be used as a proxy for global radiation, which is a critical factor influencing the local and global energy budget (Dai et al., 1999; Wild, 2012; Xia, 2013). It also has major practical implications, for example, for renewable solar energy technologies and increasing agricultural productivity (Suehrcke et al., 2013; Nishio et al., 2019).

It is clear that in the recent period, the meteorological parameters have changed for locations near the BO. Hence, the changes are evident and can be considered in subsequent analysis.

## 4 The brightness data

**Figure 3:**
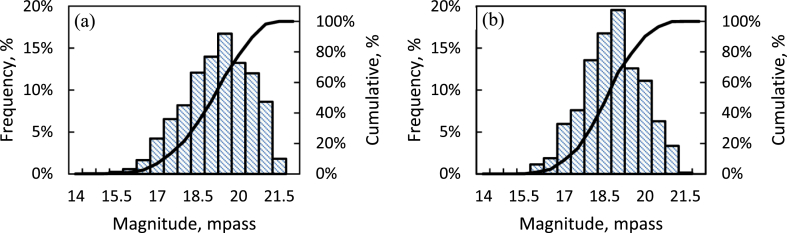
Frequency (vertical bars) and cumulative (solid line) distributions of the sky brightness for 1692 nights within the years 2011–2018 in the AM (a) and PM (b) periods. The first to third quartiles are shown in the top left corner of each panel, in units of magnitudes per arcsecond square (mpass).

Figure 3 shows the frequency distribution of all the data for the night-sky brightness during the AM and PM periods, defined as the 4 h after and before local midnight, respectively. The median brightness during the AM was 19.06 mpass, which is about 2.7% darker than the PM period, with 18.56 mpass, before correcting for the brightness of the phases of the Moon. The average brightness was 18.96 ± 1.22 mpass and 18.55 ± 1.09 mpass, respectively, for the AM and PM periods. The brightness quartiles Q1 and Q3 were also better during the AM than the PM period. The percentage of nights with skies that were darker (brighter) than the median magnitude of 19 mpass was 66% (34%) for the AM and 53% (47%) for the PM periods. However, there was even a possibility of the sky being darker than the 20^th^ magnitude, with percentages of 36% and 21% during the AM and PM periods, respectively. These favorable observational conditions in the AM period were the same as in the early history of the BO about 95 years ago (Voute, 1933).

**Figure 4:**
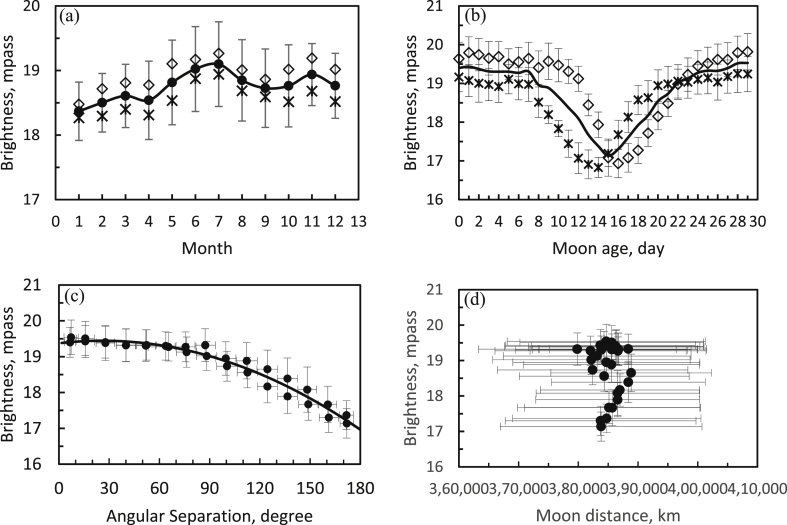
(a) Monthly average variations in sky brightness shown as filled circles with standard deviations. (b) Sky brightness as a function of the age of the Moon (in days past new Moon) shown with diamonds for the AM and with star symbols for the PM periods. (c) Brightness *vs*. the angular separation of the Sun and the Moon. (d) Sky brightness *vs.* distance to the Moon. All data are for 1 692 nights between 2011 and 2018.

Figure 4a shows the monthly average sky brightness, which varies with an amplitude of about 2 magnitudes. The yearly average value was 18.75 ± 0.22 mpass. During the AM period, the night sky was darker than during the PM period. The darkest sky occurred at the peak of the summer season, in the month of July. On the contrary, near the equinox months of April and September, the brightness magnitude was declining (*i.e*., the sky was brightening).

We can also determine the sky brightness as a function of the age of the Moon (*i.e*., the number of days past new Moon), as shown in Figure 4b. The average brightness before and after midnight have asymmetric patterns relative to the full Moon phase due to the setting and rising of the Moon. The effect of the age of the Moon causes the sky to be brighter by 3.5 magnitudes, or about 25 times brighter. Thus, the brightness of the Moon significantly affects the natural sky illumination. The AM period was consistently darker than the PM period for 4 days before and after the new-Moon phase.

The relation of the sky brightness (SB) to the angular separation *ρ* between the Sun and the Moon (see the solid lines in Figure 4c) can be represented as

(1) SB (mpass) = 19.37 ± 0.08 + 0.0054 ± 0.0022 *ρ* − 0.0001 ± 0.00001 *ρ*^2^ (R^2^ = 0.96).

In contrast, variations in the distance to the Moon exhibited no correlation with the SB, as seen in Figure 4d.

### 4.1 SB near the new-moon phase

**Figure 5:**
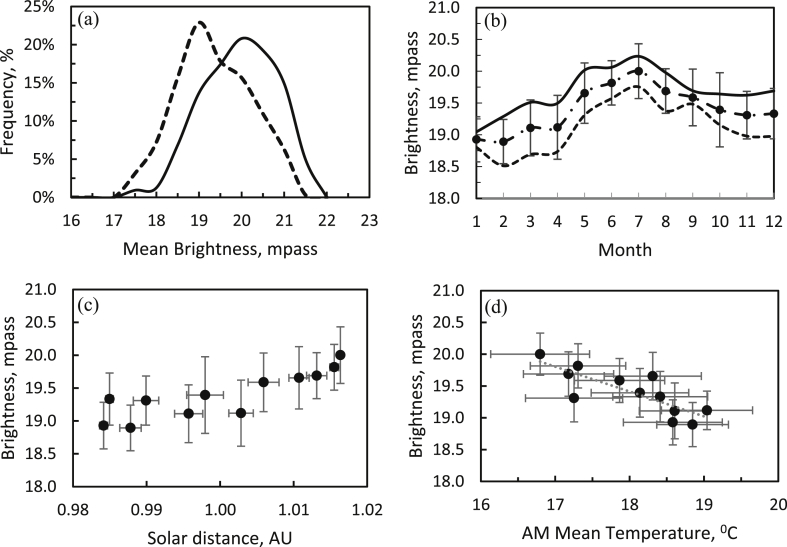
(a) The frequency distribution of sky brightness subsequent to data selection for the three days prior and subsequent to the new moon in the AM (solid line) and PM (dashed line) periods. (b) Monthly variations of the mean sky brightness (dot-dashed line), AM (solid line), and PM (dashed line) periods. (c) Variation of the mean sky brightness as a function of the solar distance. (d) The mean temperature of the AM period.

After selecting data for the 3 days before and after the new-Moon phase, we found that the percentage of nights with zenith-direction brightness larger than 19.0 mpass (*i.e*., darker skies) increased to 91% for the AM and 74% for the PM periods compared with the average. Further, the percentage of nights even darker than 20 mpass became 60% for the AM and 33% for the PM (see Figure 5a). The average brightness for the AM and PM periods on moonless nights was enhanced to the level of 19.70 ± 0.84 mpass and 19.01 ± 0.88 mpass, respectively, with median values of 19.73 mpass and 19.03 mpass (approximately 3.7% darker). That is, the sky was about 1.9 times darker than the average.

In Figure 5b, the average brightness after midnight was still darker than before midnight. Seasonal variations also affect the night-SB. Darker skies occurred in the summer season, from May to August, peaking in July, with average and maximum brightness of 20.00 ± 0.43 mpass and 20.82 mpass, respectively, in the AM period. Brighter skies occurred in February when the average brightness was 18.89 ± 0.35 mpass. The equinox in September saw the smallest difference—0.21 mpass—between the average brightness after and before midnight. However, the equinox in March showed the largest difference, 0.82 mpass. In general, the SB after July was also darker than before due to the solar position at the BO. Starting from April to December, there was a greater probability of finding a darker sky after midnight. The darkest time of night occurs at an average of 2.54 ± 0.21 h after midnight, with quartile 1, quartile 2, and quartile 3 time of 2.34, 2.57, and 2.68 h after midnight, respectively. These values have remained steady within the error bars throughout the year. As shown in Figure 2a, the average temperature in July dropped to the lowest daily value of 17.45 °C ± 1.88 °C before midnight and 16.80 °C ± 1.93 °C after midnight. These quantified seasonal variations of SB will obviously be advantageous for planning observations at the Observatory.

The periodically changing solar position in the sky also affects the night-SB. The sky becomes brighter when the Sun approaches the Earth and *vice-versa*. This also affects the duration of daylight and, consequently, is related to the duration of the night-SB. In the middle of the year in July, the shorter daylight or longer duration of nighttime corresponds to the darkest nights. The variations of the night-SB with the solar distance *d* in AU were within 1 mpass at the aphelion and perihelion positions, which is less than the brightness variation due to the phase of the Moon, as in Figure 5c. The relation between the night-SB and the solar distance is approximately linear:

(2) SB (mpass) = 25.178 ± 4.823 ∗ *d* (AU) − 5.785 ± 4.825 (R^2^ = 0.73; r = 0.85)

The Earth's elliptical orbit is closest to the Sun or perihelion in January, causing a 6% increase in the Southern Hemisphere irradiance at the top of the atmosphere around the perihelion compared to the North Hemisphere near the aphelion in July (Herman, 2010). Although the aphelion–perihelion UV variation is small, the amount of solar UV radiation reaching the ground can be enhanced because of different ozone concentrations through photolysis mechanisms. Only approximately 10% of all the atmospheric ozone is concentrated in the troposphere and less abundant than in the stratosphere. Even so, the relatively small amount of ozone in the troposphere is fundamentally important for the composition of the Earth's atmosphere. The absorption of the solar UV radiation (λ < 310 nm) by the ozone leads to the generation of electronically excited O (^1^D) atoms, which can react with water vapor to form highly reactive hydroxyl radicals. After sunset, recombination processes occur from ions excited during the day by the solar extreme ultraviolet (EUV) radiation, which will likely cause a difference in the SB before and after midnight. Many mechanisms have been proposed from products in ozone photolysis (Matsumi and Kawasaki, 2003).

### 4.2 SB, meteorological, and solar cycles

In the tropical region, the large gradient of incoming solar radiation influences the energy exchanges between the atmosphere and the surface at seasonal and diurnal cycles. The associated evaporation and convection of the energy transfer in the coupling of the surface–atmosphere influence the formation of clouds and water vapor. The convective cloud cycle is attributed to a direct thermodynamic response to the strong diurnal cycle of the land surface temperature. The night-SB is related to sky cloudiness (Herdiwijaya, 2016; Ściężor, 2020). A brighter sky is correlated with the thickness of the low cloud cover. Low-layered clouds such as the stratus and nimbostratus affect the meteorological parameter of the sunshine duration (Matuszko, 2012). As an important proxy, cloud formation connects SB to the seasonal and diurnal variations of meteorological parameters that have been extensively studied (Yang and Slingo, 2001; Stubenrauch et al., 2006).

The seasonal cycle of a high cloud amount with a minimum in July and maxima in spring and autumn is correlated with the temperature (day and night) and precipitation variations (Figure 2a and e) (Stubenrauch et al., 2006). The monthly SB follows the seasonal variation related to the temperature and precipitation profiles. The seasonal water vapor cycle, cloud amount, and atmospheric circulation influence the surface climatology with altitude dependence. However, the solar force exerted by the longer incoming radiation from the sunshine duration corresponds to the RH profile (Figure 2c and 2f). The varying seasonal phases or lag of local climatology variables do not exhibit a strong relationship with high cloud amounts (Chepfer et al., 2019).

A semi-diurnal sky-brightness cycle has been reported from several astronomical sites. Walker (1988) found a steady exponential decrease of ~0.4 mag in B and V passbands during the first half of the night at San Benito Mountain (h = 1600 m). At Kitt Peak National Observatory (h = 2096 m) and Krisciunas (1990) at Mauna Kea (h = 2800 m), Pilachowski et al. (1989) obtained a decrease of ~0.3 mpass at a certain date in the V passband in the first 6 h after the end of twilight, which is in agreement with Walker. The darkest SB was observed before the morning astronomical twilight. Pilachowski et al. (1989) also found a short time scale-varying brightness. Sky-brightness measurements were also taken by Benn and Ellison (1998)) at La Palma (h = 2360 m), Patat (2003) at ESO-Paranal Observatory (h = 2635 m), and Matilla et al. (1996) at ESO-La Silla Observatory (h = 2350 m) revealed no difference between the beginning and the end of the night and provided no support for the idea that the sky is darkest before dawn. At the BO (h = 1300 m), the median brightness during the AM was approximately 3.7% darker than the PM period. The darkest sky was observed at 2.57 am (LT). We suggest that the altitude dependence of the lower sites (h < 2000 m) gives rise to different characterizations of surface climatology and diurnal cycles compared with the higher sites (Marty et al., 2002; Wu et al., 2003; Scaff et al., 2017). Figure 5b shows a typical sign of the diurnal cycle when the temperature decreases (darker sky) and reaches a minimum in the late night. Consequently, the surface RH increases, and additional thin clouds are formed. However, opaque clouds are not always established together with RH. The mutual formation of RH with thin clouds is driven by microclimate and diurnal variation, rather than by a large-scale change in the amount of water vapor in the atmosphere. Radiative processes strongly affect the life cycle of atmospheric circulation, especially deep convective systems in the tropics. In the convective mechanism, the cold cloud coverage is dominated by spatially large, long-lived cloud systems in the near surface. They tend to form in the early evening (2–7 PM) and reach a maximum areal extent of very cold cloud tops before dawn to early morning (0–6 AM) (Chen and Houze, 1997; Hong et al., 2008; Chepfer et al., 2019).

**Figure 6:**
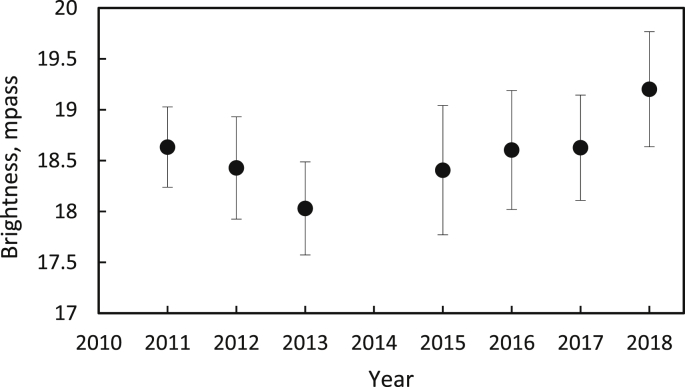
Yearly average sky brightness.

In the long-term temporal variation, SB depends on the 11-year activity cycle of the Sun (Walker, 1988; Krisciunas, 1997; Patat, 2003; Benn and Ellison, 1998; Matilla et al., 1996; Neugent and Massey, 2010). The night sky is brighter at the solar maximum phase compared with the solar minimum conditions (~0.4–1.0 mag.). We confirm this finding, as seen in Figure 6. The maximum and minimum phases of solar cycle 24 occurred in 2014 and 2018 with sunspot number ranges of 113 and 7, respectively. The SB differed by approximately 5%. The sunspot numbers and the solar radio flux at 10.7 cm were very well correlated. Both were good proxies for the solar EUV. These results reveal that the level of solar radiation in solar EUV wavelengths must be considered in the energy release from the ionized layers over some typical energy deposition time scale, as integrated through a feedback mechanism in the seasonal and diurnal cycles. When assessing the quality of a dark-sky site, the faintest apparent magnitude in the broadband V observation should be achieved in times of the solar minimum phase when the solar EUV irradiance decreases.

### 4.3 Azimuth and altitude SB

**Figure 7:**
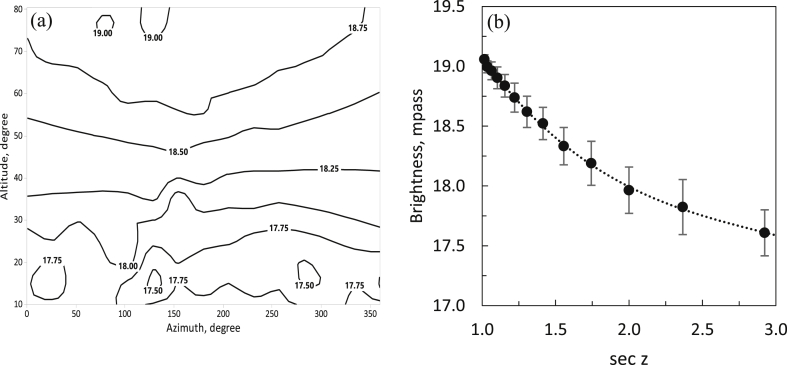
Sky brightness as a function azimuth and altitude (a) and of zenith distance (b).

On October 10, 2018, we also scanned and measured the SB as a function of azimuth and altitude every second for 1 h, starting at 19:09:18 local time. We accumulated a total of 2900 recorded data points. The starting time was just after moonset and about 1 h after sunset. Many trees obscured the horizon up to 20° (zenith distance, z = 2.924°). According to Figure 7a, light pollution can reach an altitude of 40° southward of about 160° azimuth, which is about 13 km from Bandung City. The effect from nearby Lembang City, which is 2 km away, affects the sky surrounding the observatory at altitudes of up to 30°, mostly to the east. The zenith distance of 20° was not influenced by the local sky glow.

The relation of SB to zenith distance is as follows,

(3) SB = 21.534 ± 0.200–3.398 ± 0.352 sec *z* + 1.053 ± 0.193 sec^2^*z* − 0.120 ± 0.033 sec^3^*z* (R^2^ = 0.99).

**Figure 8:**
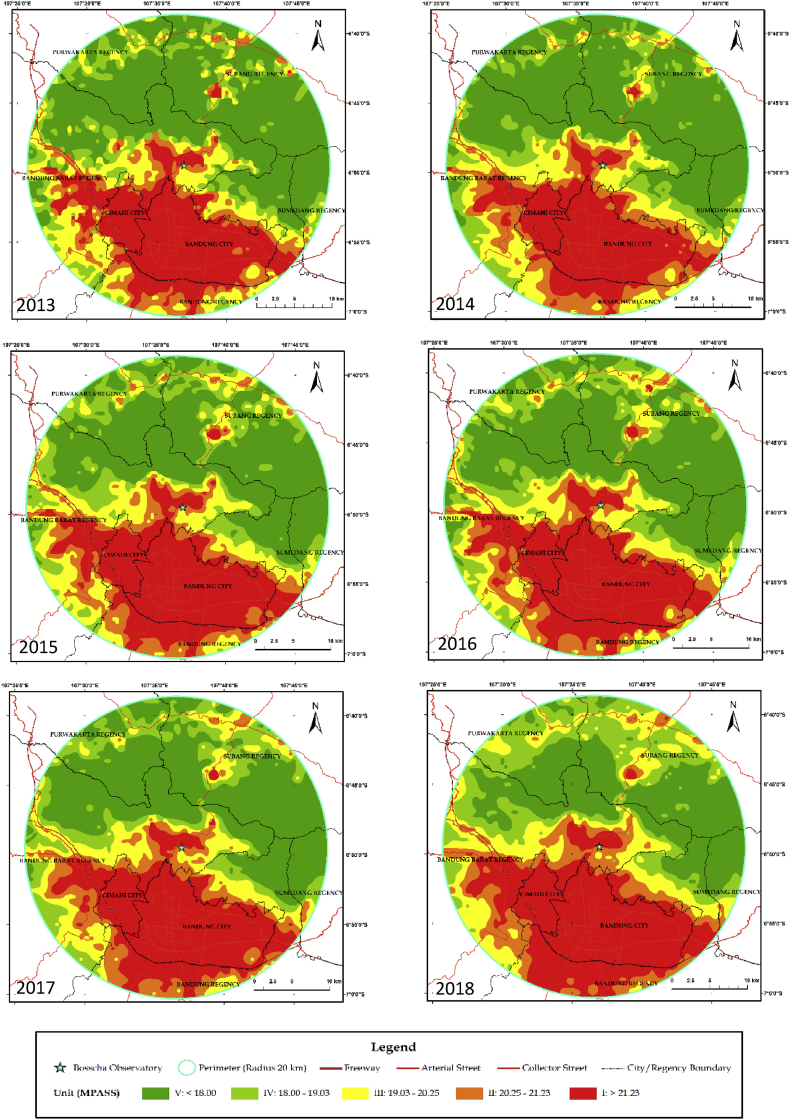
Sky-brightness distribution within a 20-km radius centered at the BO (star symbol) for 2013 to 2018.

Eq. (3) predicts a maximum SB of 19.994 mpass at the zenith. The approximate variation of air mass with zenith angle is almost the same for latitudes less than 80° (Hardie, 1962; Rozenberg, 1966; Young and Irvine, 1967 (YI 1967); Kasten and Young, 1989; Young, 1994; Pickering, 2002). One air mass is the thickness of the atmosphere in the zenith direction. The relation of the SQM magnitudes to the astronomical V Johnson magnitudes can be estimated from SQM − V = k, where k = 0.48 ± 0.12 (Cinzano, 2005) or k = 0.55 ± 0.20 (Sánchez de Miguel et al., 2017). In the V passband, the SQM has the same response as the Johnson system. We used the theoretical air mass from YI 1967 and related it to the variation of SB. In this way, we found the SQM linear extinction coefficient to be 0.77 ± 0.05 (R^2^ = 0.95). The broadband SQM transmission filters integrate over the standard U, B, and V photometric bands. The U filter corresponds to the highest extinction coefficient. At the BO, the extinction coefficient, as measured through a blue filter, increased in value from 0.5 in 1982 to 0.7 in 1993. This can be attributed to the increase in atmospheric pollutants (Malasan et al., 2001). We, thus, considered the SQM-based extinction value to be the maximum. Heavy dust particles come from high-density roads that are frequently congested with traffic within a distance of 1–2 km from the Observatory. Moreover, Lembang City is a tourist destination, with a high population density of about 2000 per km^2^ that is growing at a rate of 6%/year (1990–2010); see Figure 8.

### 4.4 Sky glows around the Bosscha Observatory

One source of sky glow comes from Bandung City, about 11 km south of the Observatory. Bandung City contributes significantly to the economy of Indonesia, with 2,470,802 citizens recorded as inhabiting a land area of 167.67 km^2^, bringing the density of Bandung City to 14.736 people/km^2^. The population growth rate of Bandung City from 2005 to 2010 was reported to be 1.15%, as the region has developed into a metropolitan area (Paramita, 2016). The Visible Infrared Imaging Radiometer Suite Day–Night Band onboard the Suomi National Polar-orbiting Partnership satellite provides cloud-free nighttime images, which can be applied for broad socio-economic purposes (Elvidge et al., 2017; Bennett and Smith, 2017; Wang et al., 2018; Proville et al., 2017). Following Zamorano et al. (2016), we converted the radiance measured by this satellite into brightness units of mpass. We then classified the brightness into five categories (see Table 1). In Figure 8, we show measured sky glow within a 20-km radius from the BO (at the center of the circle) in the month of July from 2013 to 2018. Light pollution is aligned with road lighting, which extends into the more-complex urban area. This can be seen in the east–west regions south of the Bandung Basin. At the north of the Observatory, the growth of the light-polluted area increased vastly, encircling the mountainous area to a height greater than 1000 m.

**Table 1:** Sky-brightness classifications.

Class		Typical area	Range (mpass)	Equivalent class (Crumey, 2014)
1	(Very low)	Truly dark sky	>21.30	Pristine black
2	(Low)	Rural sky	20.25–21.30	Gray
3	(Intermediate)	Suburban sky	19.03–20.24	Bright
4	(High)	Urban sky	18.00–19.02	
5	(Very high)	City sky	<18.0	White

**Figure 9:**
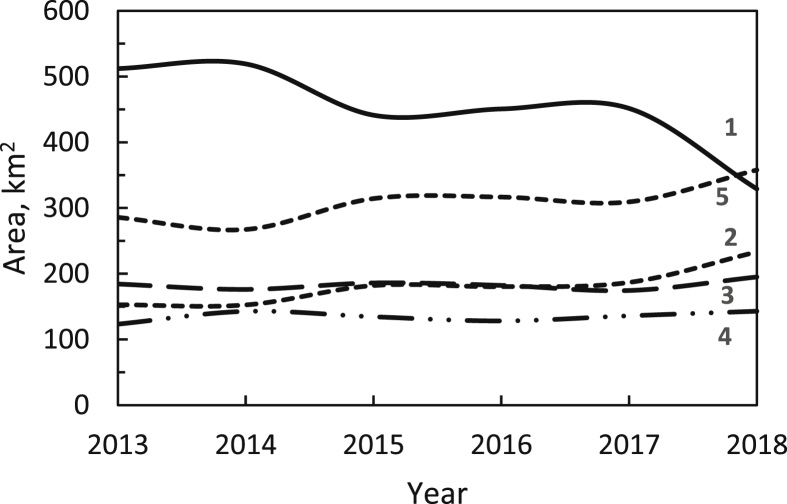
The change in the area of each class of sky brightness from the maps shown in Figure 8 (See Table 1 for definitions of the labels).

The fast growth of the cities around the Observatory caused an average decrease of 31.7 km^2^/year of class 1 SB, as can be seen in Figure 9. About 50% of the decrease in class 1 was dispersed into increases of 14.0 km^2^/year in class 5 and 14.4 km^2^/year in class 2. The average rates of the increase of classes 3 and 4 were relatively small, at 1.3 and 2.0 km^2^/year, respectively. In addition to light pollution from Bandung City to the south, the nearby northward and westward directions from the Observatory have now become disturbing sources as well. Recently, an effort has begun to cope with the growth of light pollution in Bandung Basin, starting with a presidential decree in 2018, especially for local light management within a 2.5 km radius around the BO. This is the first light pollution law in Indonesia, and law enforcement is needed to protect the night-sky darkness. A national light pollution law was subsequently developed, and it is required for the next generation of people to be able to see the natural starry night sky as human heritage.

**Figure 10:**
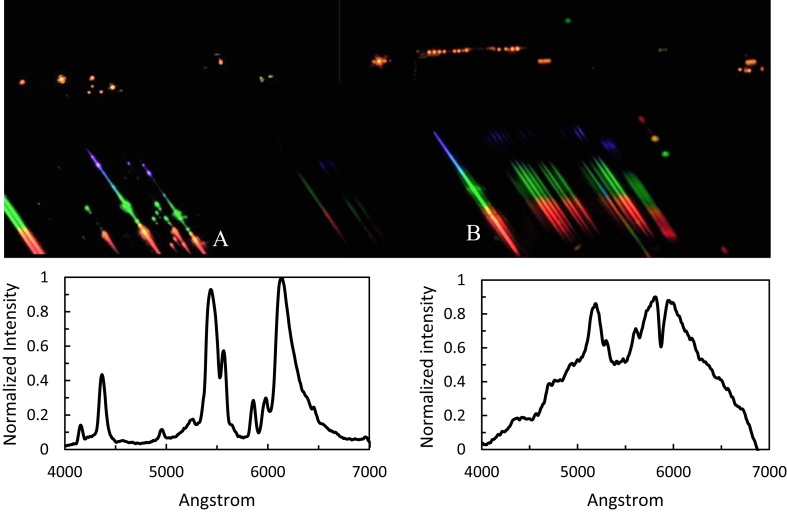
City lights spectra (top panel). Spectrum of compact fluorescent lights of group A (left) and high-pressure sodium lamps of group B (right) light sources in the southward direction toward Bandung City.

City light sources mostly belong to groups of compact fluorescent lights and high-pressure sodium lamps from streetlights (Figure 10). We identified the spectra herein with a low-resolution grating. We mounted this 200 lines/mm transmission diffraction grating in a standard 1.25″ diameter filter holder and manually attached it in front of and centered on a 50-mm camera lens. We installed the digital single-lens reflex camera and tripod on the rooftop of a seven-story building. Even from the 20-s exposure times we used, the unshielded street lighting clearly radiated upward.

## 5 Conclusions

We have measured and quantified the night-SB at BO for 1692 nights during the years 2011–2018, using a small SQM photometer. We divided each night into AM and PM time periods. Under normal conditions, the photometer provides stable measurements of SB and internal temperature. The average brightness of the AM and PM periods on moonless nights reaches 19.70 ± 0.84 and 19.01 ± 0.88 mpass, respectively, with median values of 19.73 mpass and 19.03 mpass. The maximum SB and average temperature showed consistently darker skies and cooler temperatures subsequent to midnight than prior to midnight. The darkest skies occur in the summer season from May to August, peaking in July (in the AM period), with an average and a maximum brightness of 20.00 ± 0.43 mpass and 20.82 mpass, respectively. The SB exhibits seasonal variations that depend upon the changing solar distance. However, variations in the Moon's distance had no detectable correlation with the sky-brightness variations. The brightness also depends on the temperature. We also confirm that the SB depends strongly on the age of the Moon (*i.e.,* the number of days past new Moon) and the Sun–Moon angular separation. We also found that the night-SB quality can be modified by the coupled climate system as a diurnal cycle to an 11-year solar cycle. Hence, we suggest that SB monitoring at the BO should be performed in a longer period to capture more variabilities. Moreover, considering the substantial decrease in areas with truly dark skies in recent years caused by the nearby city lights, the Observatory and the local government should collaborate in preventing the decline in night-sky quality.

## Declarations

### Author contribution statement

Dhani Herdiwijaya: Conceived and designed the experiments; Performed the experiments; Analyzed and interpreted the data; Contributed reagents, materials, analysis tools or data; Wrote the paper.

Ratna Satyaningsih: Analyzed and interpreted the data; Contributed reagents, materials, analysis tools or data; Wrote the paper.

Luthfiandaria: Performed the experiments; Analyzed and interpreted the data; Contributed reagents, materials, analysis tools or data.

Hendra A. Prastyo: Analyzed and interpreted the data; Contributed reagents, materials, analysis tools or data.

Eka P. Arumaningtyas, Maman Sulaeman, Agus Setiawan, Yuni Yulianti: Performed the experiments; Contributed reagents, materials, analysis tools or data.

### Funding statement

D. Herdiwijaya was partially supported by P3MI-Institut Teknologi Bandung 2017 Research Grant.

### Competing interest statement

The authors declare no conflict of interest.

### Additional information

No additional information is available for this paper.
